# Young Children with a Bucket-Handle Tear to the Discoid Lateral Meniscus Successfully Treated Using Arthroscopic Saucerization and Repair: Two Case Reports

**DOI:** 10.3390/medicina58101403

**Published:** 2022-10-06

**Authors:** Yu-Hsiang Liao, Chun-Ho Chen, Chii-Jeng Lin, Wei-Ren Su, Chia-Lung Shih, Chen-Hao Chiang

**Affiliations:** 1 Department of Orthopedics, Ditmanson Medical Foundation Chia-Yi Christian Hospital, Chia-Yi City 600, Taiwan; 2 Department of Orthopedics, National Cheng Kung University Hospital, Tainan City 704, Taiwan; 3 Clinical Research Center, Ditmanson Medical Foundation Chia-Yi Christian Hospital, Chia-Yi City 600, Taiwan

**Keywords:** arthroscopy, discoid meniscus, saucerization, bucket-handle tear, meniscus repairment

## Abstract

Observations of a symptomatic discoid lateral meniscus in young children are infrequent. The objective of this report was to demonstrate the use of arthroscopic saucerization and repair for treating a bucket-handle tear of a lateral discoid meniscus in two young children. Two young children (a 28-month-old girl and a 5-year-old boy) presented with a bucket-handle tear of the complete type lateral discoid meniscus. Both patients received arthroscopic saucerization and repair. A full knee extension under a long leg cast was applied for one month after surgery. The two patients were able to achieve a full range of motion of their operated knees without limping or presenting an antalgic gait at the third month after surgery. Both patients and their parents felt satisfied with the treatment at the 2- and 3-year follow-ups, respectively. Our results demonstrated that arthroscopic saucerization and repair seems to be an effective treatment for bucket-handle tears of the lateral discoid meniscus in young children—even those younger than 3 years old. We reported the youngest case (a 28-month-old girl) in comparison with the findings from a literature review.

## 1. Introduction

A discoid meniscus is a human congenital variant of the knee and is a meniscus that has an unusual shape, is thicker, and covers a larger surface of the tibial plateau than a normal meniscus. A discoid meniscus is not only a morphologic abnormality but also a histologic abnormality with reduced tissue quality [1]. The lateral meniscus is the most typical site of the abnormality, but a discoid medial meniscus is a fairly uncommon condition [2]. The most common symptoms include pain, blockage, edema, or the limitation of activities [3]. However, a person with a discoid meniscus typically does not exhibit symptoms, and the true prevalence rate is thus difficult to determine. This disease usually occurs in the lateral meniscus [3]. The prevalence of this disease varies by race, and ranges from 0.4 to 20% [4,5,6,7].

In young children, a symptomatic discoid lateral meniscus occurs less frequently. The use of arthroscopy to treat a young child with this condition is rarely reported [8]. The objective of this study was to report two young children with symptomatic discoid lateral menisci who were treated by arthroscopic management. These two cases were Watanabe type I complete lateral discoid menisci based on Watanabe’s classification [3]. To the best of our knowledge, one of them was a young girl who reported a bucket-handle tear in the lateral discoid meniscus.

## 2. Case Report

### 2.1. Case 1

**Figure 1:**
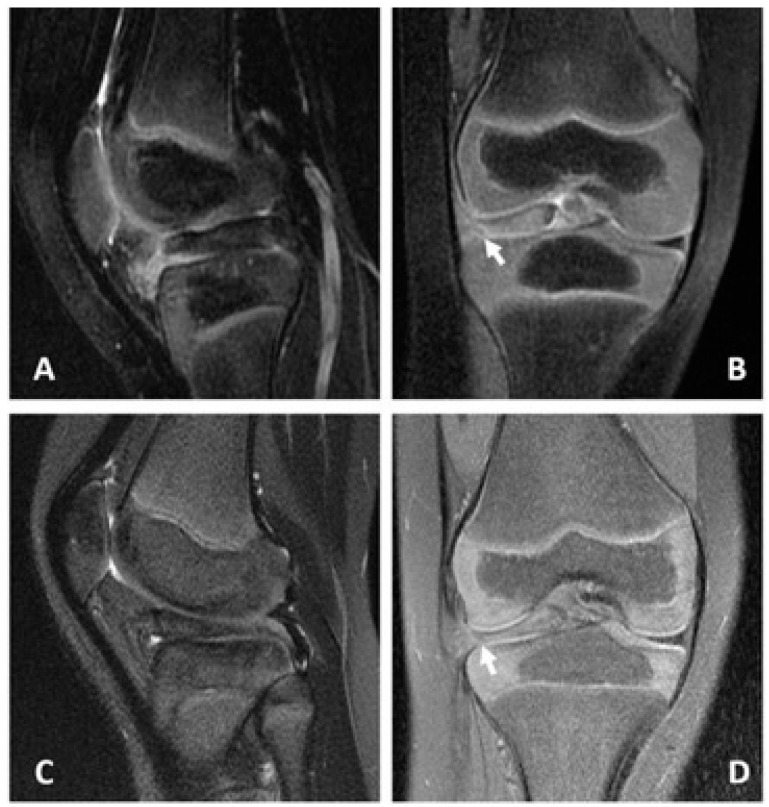
Preoperative MRI of the affected knees for the two cases. (**A**) coronal and (**B**) sagittal planes for the case of the 28-month-old girl. (**C**) coronal and (**D**) sagittal planes for the case of the 5-year-old boy. Both lateral menisci revealed increased thickness and flattening, loss of the natural bow-tie configuration, and a peripheral tear in the posterior horn. The white arrow indicates the peripheral tear site. MRI: magnetic resonance imaging.

The first case was a 28-month-old female toddler who was a term baby without hereditary diseases. A mildly limping gait and a loss of full the extension of her right knee were discovered about one month after she experienced a fall. The range of motion (ROM) of her affected knee was from 10° to 120°. There were no local signs of infection or instability, and no palpable masses in the affected knees were found after physical examination. The weight-bearing radiographies demonstrated no bony deformities over the knee and hip. An increased thickness and flattening of the lateral meniscus, a loss of the normal bow-tie configuration, and a peripheral tear over the posterior horn were all seen upon magnetic resonance imaging (MRI), indicating that this instance demonstrated a full discoid lateral meniscus with a bucket handle tear (Figure 1A,B).

**Figure 2:**
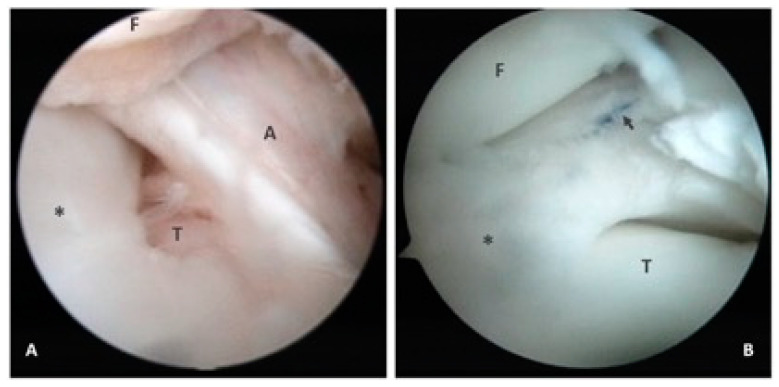
Arthroscopic findings for the 28-month-old toddler. A complete discoid lateral meniscus was found after reduction (**A**). A bucket-handle tear to the lateral meniscus was repaired by employing inside-out techniques using No. 0 PDS II sutures (Ethicon, Johnson & Johnson, Somerville, MA, USA) after saucerization (**B**). A—anterior cruciate ligament; T—lateral tibial plateau; F—lateral femoral condyle; *—lateral meniscus; black arrow—meniscus repaired using No. 0 PDS II sutures.

The patient received arthroscopic management. Arthroscopy was performed under general anesthesia through the anteromedial and anterolateral portals. A small arthroscopy (2.9 mm, ConMed, Utica, NY, USA) with 30 mmHg of normal saline fluid pressure was employed due to the small knee joint. On the diagnostic arthroscopy, a dislodged lateral meniscus with an anterior impingement was found. After reduction, a complete discoid meniscus was found (Figure 2A). The patient subsequently received saucerization, which was started at the central free edge of the meniscus, excising from back to front using basket punches (Smith & Nephrew, London, UK) or a shaver (2.9 mm, ConMed, Utica, NY, USA) to restore the normal shape of the lateral meniscus. The patient underwent an outside-in meniscal repair technique using No. 0 PDS II sutures (Ethicon, Johnson & Johnson, Somerville, MA, USA) because of instability during knee flexion of more than 120° after saucerization (Figure 2B). After repair, a long leg cast was adopted to maintain full knee extension for one month.

**Figure 3:**
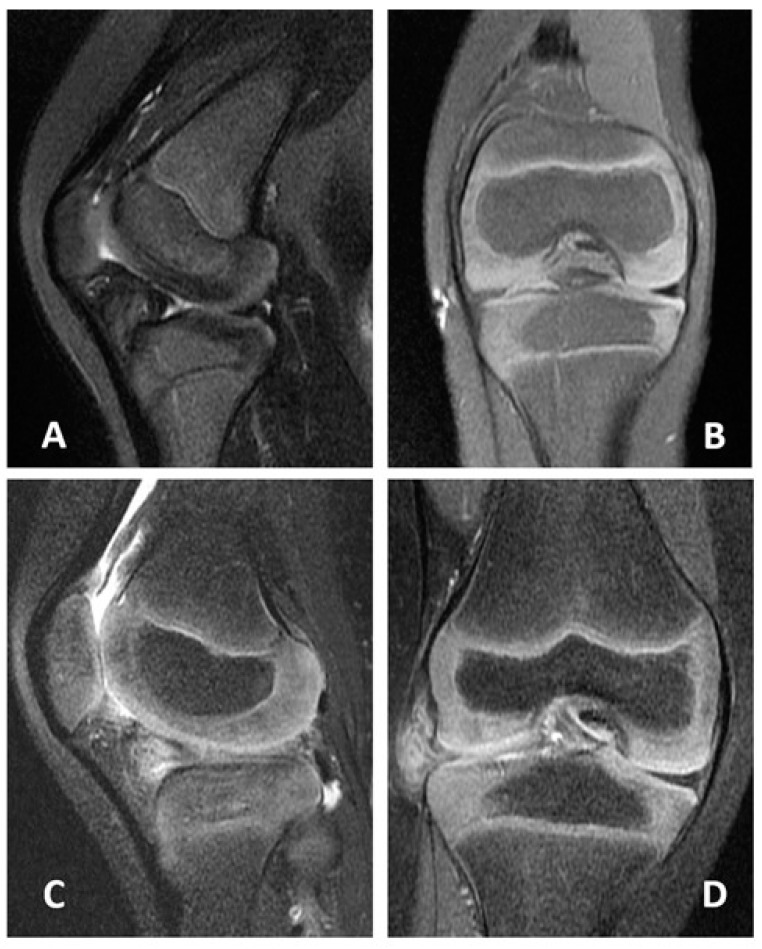
MRI of the affected knees for the two cases at the 6-month follow-up. (**A**) coronal and (**B**) sagittal planes for the case of the 28-month girl. (**C**) coronal and (**D**) sagittal planes for the case of the 5-year-old boy. A triangular lateral meniscus without a retear was found in both cases. MRI—magnetic resonance imaging.

On the first day following surgery, the patient was discharged from the hospital. Together with a lengthy leg cast, the full weightbearing of the operated knee was allowed immediately after surgery. One month following surgery, the cast was removed, and a full ROM was attempted. The patient was able to achieve a complete ROM of her operated knee at the 3-month follow-up, and there was no limping or antalgic gait. At six months following surgery, MRI imaging revealed no retear of the lateral meniscus (Figure 3A,B). At the 35-month follow-up, the patient and her parents were satisfied with the treatment (Supplementary Figure S1).

### 2.2. Case 2

The second case, a 5-year-old boy, was healthy before visiting our clinic. Pain and difficulty fully extending the right knee manifested about six months after a twisting injury. The ROM of his right knee ranged from 5° to 110°. A physical examination revealed no local symptoms of infection or instability and no palpable lumps in the affected knee. MRI revealed an increased thickness and flattening of the lateral meniscus, a loss of the natural bow-tie configuration, and a peripheral tear over the posterior horn, which signaled that this patient had a complete discoid lateral meniscus with a bucket handle tear (Figure 1C,D).

The same treatment as that of case 1 was applied to this patient. Following reduction, a full discoid meniscus was discovered, and he subsequently received saucerization. Due to instability during knee flexion of more than 60° following saucerization, the patient underwent an inside-out meniscal repair utilizing two No. 0 PDS II sutures. After the repair, a one-month long leg cast was used to maintain complete knee extension.

The patient was discharged from the hospital on the first day after surgery. Full weightbearing of the operated knee was allowed immediately after surgery alongside the implementation of a long leg cast. The cast was removed at one month after surgery, and the patient was asked to train the full ROM of the knee. At the 3-month follow-up, the patient was able to achieve a full ROM of his operated knee, and there was no limping or antalgic gait. MRI images demonstrated no retear of the lateral meniscus at six months following surgery (Figure 3C,D). No limping gait was present, and his parents felt satisfied at the 25-month follow-up.

## 3. Discussion

Young children with a discoid lateral meniscus are rarely reported, especially in the case of those under 6 years old. Several young cases have been reported in the literature. Nathan et al. reported a 4-month-old child with a lateral discoid meniscus via a physical examination and pathologic findings [9]. For a 6-month-old child, Barnes et al. used computed tomography and MRI to identify a lateral discoid meniscus [10]. The youngest patient receiving arthroscopic saucerization for a torn discoid lateral meniscus was a 26-month-old girl [8]. The efficacy of arthroscopic saucerization for treating young patients with discoid lateral menisci is still unclear. In this study, we reported two young children (a 28-month-old and a 5-year-old) who had a complete type discoid lateral meniscus with a bucket-handle tear, and they were successfully treated using arthroscopic saucerization and repair.

For a young child with knee pain, swelling, and a limited range of motion or a limping gait, there are possible severe diseases in which a differential diagnosis is required, such as discoid menisci, popliteal cysts, osteochondritis dissecans, patellofemoral instability, patellofemoral pain syndrome, Osgood–Schlatter disease, Sinding–Larsen–Johannson disease, Blount’s disease, infection, trauma, etc. [11]. Therefore, we arranged the radiographies and MRI to perform the differential diagnosis for our young case, who showed a full discoid lateral meniscus with a bucket handle tear along with incomplete knee extension.

The biomechanical differences after receiving arthroscopic partial meniscectomy for patients with discoid lateral meniscus injuries have been investigated [12]. Before surgery, the discoid lateral meniscus group had worse outcomes, including a shorter stride length, a lower walking speed, a lower maximum knee flexion during a stance phase and swing phase, a smaller adduction–abduction ROM during the gait cycle, and a lower first peak knee flexion moment compared with the healthy group [12]. After surgery, the results demonstrated that no differences were observed between the discoid lateral menisci and the healthy groups with respect to these parameters [12]. This indicates that meniscectomy is an effective treatment for discoid lateral meniscus injuries.

The size of the arthroscopy adopted for surgery is dependent on different body parts. For a knee arthroscopy, a 4-mm diameter arthroscopy is generally adopted. For a small joint arthroscopy, the use of an arthroscope with a diameter of less than 3 mm is suitable, such as a wrist. We chose an arthroscopy with a small size (2.9 mm in diameter) for our cases because their knee joints were small.

In the past, a complete meniscectomy was the only option for treating a symptomatic discoid meniscus in young patients. Nowadays, saucerization and repair have replaced the complete meniscectomy due to a potentially high risk of long-term osteoarthritic changes [13,14]. Repairment has been suggested for the clinical presentation of instability after saucerization [13], especially for a bucket-handle tear in the discoid meniscus. To date, only a few young cases with a discoid meniscus receiving saucerization and repair have been reported. Fazio et al. reported a 32-month-old boy who received arthroscopic saucerization and anterior and posterior horn repair for an atypical, hypermobile discoid lateral meniscus [15]. The youngest case—with a bucket-handle tear in the lateral discoid meniscus that underwent arthroscopic saucerization and repair—was a 3-year-old patient [14]. Although many young cases with a bucket-handle tear in the lateral discoid meniscus treated by arthroscopic saucerization and repair have been reported, the youngest case was 3.1 years old [16]. To our best knowledge, the 28-month-old girl in this study was the youngest reported case to receive arthroscopic saucerization and repair for a bucket-handle tear of the lateral discoid meniscus, where the short-term results were satisfactory. This indicates that arthroscopic saucerization and repair seems to be effective for young patients (<3 years old) with a bucket-handle tear of the discoid lateral meniscus.

These two patients had received saucerization with meniscal repair. From our literature review, the restriction of the knee’s motion with a hinged brace was suggested. However, since these two patients are too young, using a hinged knee brace is worse than using a long leg cast to improve compliance. Therefore, long leg casts were applied for the first month after surgery. Otherwise, the rehabilitation protocol was not different from the suggestions by the previous studies [17,18].

There are some limitations of this report. Although our results demonstrated that arthroscopic saucerization and repair could be used to treat young children with bucket-handle tears to a discoid lateral meniscus, the follow-up periods for these two cases were short. Moreover, only two cases were reported, and more cases were required to confirm the effects of our treatment.

## 4. Conclusions

It is rare to observe a symptomatic discoid lateral meniscus in young children under 6 years old. Here, we reported two young cases who had a complete discoid lateral meniscus with a bucket handle tear. Nowadays, a complete meniscectomy has been replaced by saucerization and repair due to a potentially high risk of long-term osteoarthritic changes. Only a few young cases who received arthroscopic saucerization and repair were reported, and the efficacy for young cases is still uncertain. In this study, we reported two young cases with discoid lateral menisci, and they were successfully treated by arthroscopic saucerization and repair. Presently, one of our two cases is the youngest child to have received arthroscopic saucerization and repair. Our results demonstrated that arthroscopic saucerization and repair can be used to treat young children with bucket-handle tears to a discoid lateral meniscus, even for a 28-month-case. However, the follow-up periods for these two cases were short; thus, the clinical outcomes of these cases should be observed for a longer period of time. More young cases are required to further confirm the clinical outcomes after receiving the treatment.

## Data Availability

All data were mentioned in the text.

## Supplementary Materials

The following supporting information can be downloaded at: https://www.mdpi.com/article/10.3390/medicina58101403/s1, Figure S1: Pictures of the affected knee for the 28-month-old toddler at the 1-year follow-up. Full extension of the right knee was achieved (A). Arthroscopic wound was well healed (B).

## Abbreviations

ROM range of motion.

## References

[1] Tudisco C., Botti F., Bisicchia S. (2019). Histological Study of Discoid Lateral Meniscus in Children and Adolescents: Morphogenetic Considerations. Joints.

[2] Yang X., Shao D. (2019). Bilateral discoid medial Meniscus: Two case reports. Medicine.

[3] Saavedra M., Sepúlveda M., Jesús Tuca M., Birrer E. (2020). Discoid meniscus: Current concepts. EFORT Open. Rev..

[4] Ahn J.H., Shim J.S., Hwang C.H., Oh W.H. (2001). Discoid lateral meniscus in children: Clinical manifestations and morphology. J. Pediatric. Orthop..

[5] Yaniv M., Blumberg N. (2007). The discoid meniscus. J. Child. Orthop..

[6] Smillie I.S. (1948). The congenital discoid meniscus. J. Bone Jt. Surg. Br. Vol..

[7] Ikeuchi H. (1982). Arthroscopic treatment of the discoid lateral meniscus. Tech. Long-Term Results Clin. Orthop. Relat. Res..

[8] Lee B.-I., Choi H.-S. (2003). Arthroscopic treatment of symptomatic discoid lateral meniscus in a 26-month-old girl. Arthrosc. J. Arthrosc. Relat. Surg..

[9] Pa N., Sc C. (1969). Discoid meniscus. a clinical and pathological study. Clin. Orthop..

[10] Barnes C.L., McCarthy R.E., VanderSchilden J.L., McConnell J.R., Nusbickel F.R. (1988). Discoid Lateral Meniscus in a Young Child: Case Report and Review of the Literature. J. Pediatric Orthop..

[11] Orth R.C. (2013). The pediatric knee. Pediatric. Radiol..

[12] Li Y., Wu Y., Zeng Y., Gu D. (2020). Biomechanical differences before and after arthroscopic partial meniscectomy in patients with semilunar and discoid lateral meniscus injury. Am. J. Transl. Res..

[13] Davidson D., Letts M., Glasgow R. (2003). Discoid meniscus in children: Treatment and outcome. Can. J. Surg..

[14] Good C.R., Green D.W., Griffith M.H., Valen A.W., Widmann R.F., Rodeo S.A. (2007). Arthroscopic treatment of symptomatic discoid meniscus in children: Classification, technique, and results. Arthrosc. J. Arthrosc. Relat. Surg. Off. Publ. Arthrosc. Assoc. N. Am. Int. Arthrosc. Assoc..

[15] Fazio M.G., Royston E.J., Rooks V.J. (2013). An atypical symptomatic discoid lateral meniscus in a toddler. Radiol. Case Rep..

[16] Yoo W.J., Jang W.Y., Park M.S., Chung C.Y., Cheon J.E., Cho T.J., Choi I.H. (2015). Arthroscopic Treatment for Symptomatic Discoid Meniscus in Children: Midterm Outcomes and Prognostic Factors. Arthrosc. J. Arthrosc. Relat. Surg. Off. Publ. Arthrosc. Assoc. N. Am. Int. Arthrosc. Assoc..

[17] Kocher M.S., Logan C.A., Kramer D.E. (2017). Discoid Lateral Meniscus in Children: Diagnosis, Management, and Outcomes. J. Am. Acad. Orthop. Surg..

[18] Feroe A.G., Hussain Z.B., Stupay K.L., Kocher S.D., Williams K.A., Micheli L.J., Kocher M.S. (2021). Surgical Management of Medial Discoid Meniscus in Pediatric and Adolescent Patients. J. Pediatric. Orthop..

