# Supplementary material for: Young Children with a Bucket-Handle Tear to the Discoid Lateral Meniscus Successfully Treated Using Arthroscopic Saucerization and Repair: Two Case Reports
Source: Medicina (Kaunas). 2022 Oct 6;58(10):1403. doi: 10.3390/medicina58101403 (PMC9607120; doi:10.3390/medicina58101403)
Supplement: Supplementary file 1 [file medicina-58-01403-s001.zip › medicina-1923142-supplementary.pdf]

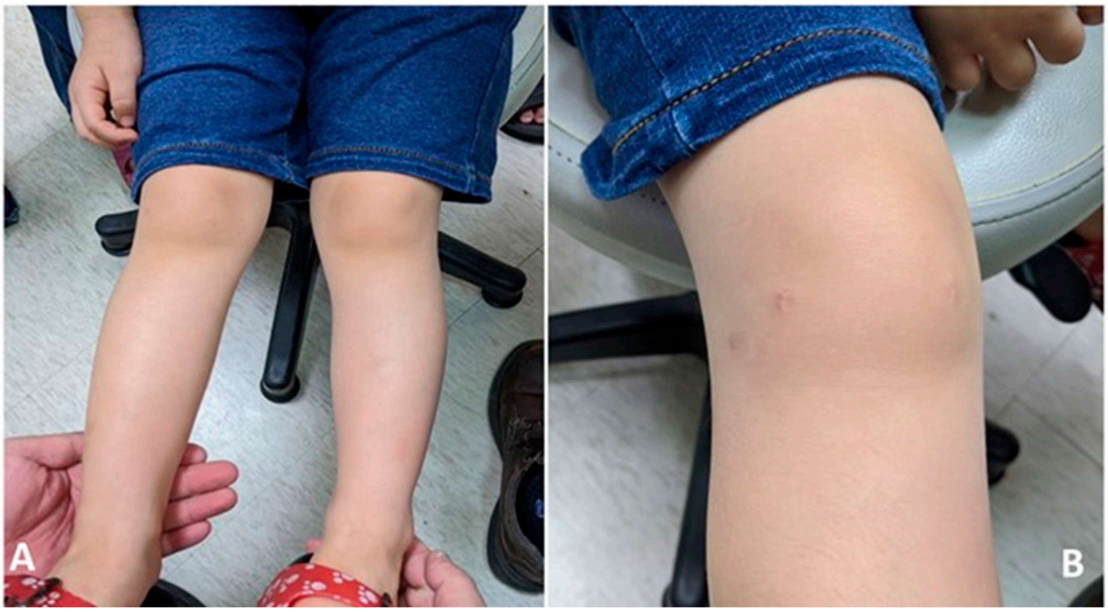

Figure S1. Pictures of the affected knee for the 28-month-old toddler at the 1-year follow-up. Full extension of the right knee was achieved (A). Arthroscopic wound was well healed (B).
